# Relapsed/Refractory Mantle Cell Lymphoma: Beyond BTK Inhibitors

**DOI:** 10.3390/jpm12030376

**Published:** 2022-03-01

**Authors:** Madelyn Burkart, Reem Karmali

**Affiliations:** 1 Feinberg School of Medicine, Northwestern University, Chicago, IL 60611, USA; 2 Division of Hematology/Oncology, Robert H. Lurie Comprehensive Cancer Center, Northwestern University, Chicago, IL 60611, USA; reem.karmali@northwestern.edu

**Keywords:** mantel cell lymphoma, relapsed/refractory mantle cell lymphoma, BTK inhibitors, CAR-T cells

## Abstract

Mantle cell lymphoma (MCL) is a rare mature B-cell non-Hodgkin lymphoma (B-NHL) with historically poor outcomes. Virtually all patients will eventually experience refractory or relapsed (R/R) disease, with a virulent course of resistance and serial relapses, making treatment challenging. The available therapies for R/R MCL are not curative with conventional therapy, their goal being to palliate and prolong survival. A variety of agents approved for R/R MCL, including Bruton’s tyrosine kinase inhibitors (BTKi), changed the treatment landscape of R/R MCL. In the pre-BTKi era, the median progression-free survival (PFS) in R/R disease was 4–9 months. With the introduction of ibrutinib, the median PFS improved to 13–14.6 months. Despite these impressive results, the duration of response is limited, and resistance to BTKi inevitably develops in a subset of patients. Outcomes after progression on BTKi are extremely poor, with a median overall survival (OS) of 6 to 10 months. Certain therapies, such as chimeric antigen receptor (CAR) T cells, have shown promising results after BTKi failure. The preferred combination and sequencing of therapies beyond BTKi remain unestablished and are currently being investigated. In this review, we describe the current evidence for the available treatment of R/R MCL after progression on BTKi.

## 1. Introduction

Mantle cell lymphoma (MCL) is a rare and aggressive mature B-cell non-Hodgkin lymphoma (B-NHL) with historically poor long-term survival rates. In western countries, MCL comprises about 7% of adult-onset NHL [1,2]. The incidence increases with age, with a median age of diagnosis of 68 years [3]. MCL is characterized by the t(11;14) (q13; q32) translocation, which leads to the overexpression of cyclin D1. The disease is heterogeneous, with some patients presenting with aggressive disease and others with a more indolent course. MCL remains an incurable disease with conventional therapy, resulting in serial relapses, making treatment extremely challenging. Survival depends on initial prognostic factors and high-risk disease features, such as a high mantle cell lymphoma international prognostic index (MIPI) score, elevated Ki-67, TP53 mutations, del 17p, complex karyotype and blastoid/pleomorphic variants. Patients with high-risk disease have inferior survival rates compared to patients without high-risk MCL; however, long-term survival remains poor for all, with a median time to treatment failure of 4.1 years and 7.7 years, depending on initial treatment with R-CHOP or R-DHAP, respectively, and a 10-year overall survival (OS) rate of 55–60% [4,5]. 

The current standard of care for advanced MCL is based on patient age and comorbidities. Younger, fit patients are generally treated with three components: cytarabine-containing induction chemotherapy, followed by autologous stem cell transplant (ASCT) and rituximab maintenance; while less fit patients receive less intensive chemoimmunotherapy without ASCT, such as bendamustine-rituximab (BR) with or without cytarabine, followed by rituximab maintenance therapy [4,6,7]. Despite the initial response rates of 50–70% with these upfront regimens, the median progression-free survival (PFS) remains only 7 to 10 years as patients will eventually progress after treatment, resulting in successive relapses each with shorter periods of remission, each being more challenging to treat [3,5,8]. 

Additionally, it is clear that patients with high-risk disease features derive minimal benefit from induction with conventional cytarabine-based immunochemotherapy regimens, including HCT. Patients with a combined high MIPI and Ki-67 expression score were shown to have a median OS of only 2 years. Patients with TP53-mutated MCL are also known to have inferior outcomes and are associated with high ki-67% and blastoid morphology, with a significantly shorter median OS of 13 months compared to 43 months seen in wild-type TP53 [9]. Patients with the blastoid or pleomorphic variants, which comprise less than 20% of cases, suffer from what are among the most aggressive subsets of MCL. Clinical trials investigating conventional chemoimmunotherapy, followed by ASCT, showed that patients with the blastoid variant had a significantly inferior 5-year OS rate of 38%, compared to 75% in patients with classical MCL [10]. Patients with high-risk features are also at increased risk of developing CNS disease, which is a rare yet serious condition, and are known to have a high mortality rate, with a median survival of only 3.7 months [11]. This poor response to frontline therapy suggests that novel treatment strategies are needed for these groups.

Recent progress in understanding the biology of MCL has led to the development of a number of novel targeted therapies, and the treatment landscape for MCL continues to evolve. The introduction of Bruton’s tyrosine kinase (BTK) inhibitors has shown promising results in survival outcomes in relapsed/refractory (R/R) MCL and is the primary choice of treatment, especially in patients in the early stages of the disease. Ibrutinib was shown to be effective for R/R disease, with an overall response rate (ORR) of 68% when used as a single agent, and ORRs of 71% and 88% when ibrutinib was combined with venetoclax and rituximab, respectively [12,13]. However, many patients will inevitably experience progression on BTK inhibitors (BTKi), which is associated with poor outcomes and a median OS of only 6 to 10 months [14,15,16]. Several agents have been investigated after BTKi failure, including combination therapies and new cellular therapies. However, the optimal approach to treating R/R disease after BTKi failure remains to be defined, and challenges in the treatment of R/R MCL persist. The goal of this review is to provide an overview of the current evidence for the available treatment approaches in R/R MCL after BTKi failure. 

## 2. BTK Inhibitors in MCL

BTK is a non-receptor kinase downstream from the B cell receptor (BCR); it is critical for normal B cell maturation, proliferation and survival. The emergence of BTKi has had a great impact on patient outcomes in the past decade, including those patients who were previously heavily treated. Ibrutinib is a small molecule inhibitor of BTK and is the most commonly used single agent for R/R MCL. The landmark international phase-II study of ibrutinib monotherapy, at a dose of 560 mg once daily in patients previously treated with bortezomib, demonstrated an unprecedented response, with a 17.5-month PFS, overall response rate (ORR) of 67%, and a complete response (CR) rate of 23% [3]. Compared to temsirolimus, ibrutinib improved the PFS from 6 to 16 months in the R/R setting and had a trend toward improved OS (30 months vs. 24 months) [17,18]. In this clinical trial, ibrutinib was relatively well tolerated and was associated with fewer treatment-related adverse effects than temsirolimus. However, the rate of grades 3 and 4 adverse events related to bleeding and atrial fibrillation was higher in the ibrutinib group (9% and 5%, respectively) [18]. These toxicities can be attributed to the numerous off-target effects of ibrutinib, which led to the development of more targeted next-generation BTKi, with the goal of improving efficacy and reducing toxicities. 

Acalabrutinib is a potent second-generation BTKi that is more selective than ibrutinib, resulting in less off-target activity and fewer toxicities. The FDA granted accelerated approval of acalabrutinib in 2019 in MCL patients who had received at least one prior therapy, based on the phase-II study, ACE-LY-004 [19]. An extended follow-up analysis of a median of 26 months demonstrated an ORR of 81%, with a CR of 43% and partial response (PR) of 38%, and a PFS of 20 months [20]. Acalabrutinib not only demonstrated a durable response in patients with R/R disease but also had a favorable safety profile, with few discontinuations due to adverse events (8%) [20].

Zanubrutinib, a next-generation irreversible BTKi, was also approved in 2019 by the FDA for R/R MCL, based on the results of the phase-II BGB-3111-206 and phase-I/II BGB-3111-AU-003 trials in R/R MCL [21,22,23]. The primary efficacy outcome measure in both trials was ORR. In the BGB-3111-206 trial, the ORR was 84%, with a CR of 59% and a median duration of response (DOR) of 19.5 months. The ORR was consistent in all subgroups, including patients with high MIPI scores and with the blastoid variant [22,23,24]. In the BGB-3111-AU-003 trial, the ORR was 84%, with a CR of 22% and a median DOR of 18.5 months [21]. Zanubrutinib was shown to be well tolerated, with the most common side-effects being neutropenia (16.3%), lung infection/pneumonia (5.8%), thrombocytopenia (4.7%), and major bleeding, reported in only 2.3% of patients. No cases of atrial fibrillation were reported, and only 10.5% of patients reported adverse reactions leading to treatment discontinuation [22,23,24]. 

Although the response rates with single-agent BTKi are very promising and relatively high, nearly all patients will eventually progress within 18–24 months and have relatively poor prognoses, with a median OS of between 6 and 10 months, despite salvage therapies [25]. Of these patients, about one-third develop progression due to primary BTKi resistance, while others have primary refractory disease based on aggressive features or discontinue treatment due to off-target toxicities and develop noncompliance. Among 20 patients with TP53 mutations treated with ibrutinib, the ORR was 55%, the median PFS was 4 months, and the median OS was only 12 months [26]. Responses to single-agent BTKi treatment in patients with TP53 mutations are brief, and alternative treatment approaches are needed for this high-risk population.

Ibrutinib, acalabrutinib and zanubrutinib are all covalent BTKi. An overview of the data for first- and second-generation BTK inhibitors in R/R MCL is summarized in Table 1. Resistance to these BTKi in MCL is often related to point mutations affecting the BTKi binding site, cysteine 481 (C481), and is less commonly related to other mechanisms of resistance, such as activating mutations in the nuclear factor kB (NF-kB) pathway and gain-of-function mutations in the downstream signaling substrates, such as phospholipase Cy2 (PLCy2) [27]. Resistance can develop during ibrutinib treatment, as well as with acalabrutinib and zanubrutinib. There are multiple resistance patterns now being studied in patients treated with BTKi, and there is still more to learn regarding therapeutic strategies to prevent resistance from developing. In an effort to address progression due to resistance to these “traditional” BTKi, there are new BTKi, as well as other agents, currently in development. 

## 3. Noncovalent BTK Inhibitors

Pirtobrutinib, or LOXO-305, is a highly selective, non-covalent next-generation BTKi demonstrating the potent inhibition of both wild-type BTK and C481 mutant BTK [28]. The BRUIN phase-I/II study demonstrated the promising efficacy of pirtobrutinib in B cell malignancies. The study included 323 patients: 61 evaluable patients with MCL had received a median of three prior therapies; 93% had prior covalent BTKi exposure. The ORR was 52% in all MCL patients, with a 25% CR rate. In patients with prior exposure to BTKi, the ORR was 52%, and it was 60% for those patients without prior exposure (including all disease types) [29,30,31]. In 20 patients harboring the C481 mutation, the ORR was 75% [29]. Pirtobrutinib was relatively well tolerated; 87% of the adverse events reported were grade 1–2 and 10% of the adverse events reported were grade ≥ 3 AE, the most common being neutropenia [31]. The durability of response of pirtobrutinib remains unknown and longer follow-up data are needed. 

There are several other non-covalent BTKi in preclinical and/or early clinical development that may offer a therapeutic approach to overcome covalent BTKi failure and resistance in R/R MCL. An example is nemtabrutinib (also known as MK-1026, formerly known as ARQ-531), a reversible dual inhibitor of wild-type BTK and the C481 mutant BTK. Preclinical results show that ARQ-531 had better survival compared to ibrutinib in chronic lymphocytic leukemia (CLL), diffuse large B cell lymphoma (DLBCL) and Richter’s transformation mouse models, and was able to inhibit the activation of both the C418 mutant and PLCy2 mutants in CLL cells, demonstrating activity against BTKi resistance [32]. The phase-I dose-escalation study evaluating ARQ-531 in R/R B cell malignancies demonstrated not only a manageable safety profile but also an anti-tumor effect in heavily pretreated patients, with 14 of 47 patients achieving a PR. Only one patient with MCL was included [33]. The ongoing phase-II study includes 118 patients (6 with MCL) and showed promising results in CLL/small lymphocytic lymphoma (SLL) [33,34]. The ongoing clinical development of MK-1026 in R/R B cell malignancies will hopefully include a larger MCL population, so as to better understand its role in the treatment of this disease (NCT03162536).

## 4. Beyond BTK Inhibitors: Targeted Therapies 

Patients who progress while receiving BTKi therapy typically have aggressive disease with relatively short survival. Treatment beyond BTKi remains unclear, and although agents have shown activity in this setting, the DOR is poor and consolidation with allogeneic hematopoietic cell transplantation (HCT) or other emerging cellular therapy approaches should be considered. It should also be noted that some of the trials did not include patients previously exposed to BTKi; therefore, their value as a treatment after BTKi is uncertain. 

Other targeted agents have previously been shown to have clinical activity in R/R MCL, including the approved agents, bortezomib, lenalidomide, and temsirolimus. Bortezomib, a proteasome inhibitor, received FDA approval in R/R MCL, based upon phase-II clinical data demonstrating a median PFS of approximately 6 months and the landmark PINNACLE trial [35,36]. Temsirolimus was approved in Europe after a phase-III trial demonstrated its superiority with a median PFS of 4.8 months compared to the investigators’ choice [37]. Lenolidomide is a second-generation immunomodulator that has been studied extensively and demonstrated efficacy in R/R MCL prior to the introduction of BTKi [38,39]. However, these drugs have not shown promising results after BTKi failure. The observational MCL-004 study showed that lenolidomide (including monotherapy and in combination with rituximab or other agents) had a 27% ORR and 14% CR, with a median DOR of 18 weeks in patients who had previously failed on ibrutinib. Patients treated solely with lenalidomide monotherapy (N = 13) had an ORR of 15%, with no patients achieving a CR [40]. There are also no clinical trials that have studied the other aforementioned agents used alone beyond BTKi treatment and, given their inferior response compared to BTKi alone, they have limited utility as single-agent therapies and are likely best used in combination strategies in this setting. 

About 90% of MCL cases overexpress B-cell lymphoma 2 (Bcl-2), which plays a role in protecting MCL cells from apoptogenic signals. Venetoclax, a Bcl-2 inhibitor, has shown single-agent activity in a phase I study in patients with MCL without prior BTKi treatment, and limited data regarding the efficacy of venetoclax post-BTKi therapy [41]. There is a small report of 20 patients with R/R MCL and prior BTKi treatment who were treated with venetoclax alone in the United Kingdom, with a 53% ORR and 18% CR; however, the median PFS was only 3 months [42]. Another retrospective study of 24 R/R MCL patients, 22 of whom had prior BTKi failure, achieved an ORR of 50% and a CR of 21% with venetoclax; however, only half of the patients received single-agent venetoclax [43]. More research has focused on combining the two agents, due to the synergistic relationship between BTKi and venetoclax. 

Notably, in the AIM trial exploring venetoclax with ibrutinib, TP53 aberrations were present in 50% of the patients. Despite this finding, the ORR was 71%. Half of the patients with TP53 aberrations achieved a CR, and five out of six patients with confirmed ibrutinib resistance achieved a CR. The median PFS was 29 months. The OS rate was 79% at 12 months and 74% at 18 months [13]. As expected, the reported rates of all-grade toxicities were higher than those seen when ibrutinib was used alone. The phase-III SYMPATICO trial is currently exploring the long-term benefits of venetoclax in addition to ibrutinib therapy over ibrutinib alone. In this study, venetoclax was administered concurrently with ibrutinib, unlike in the AIM trial, where patients received a 4-week ibrutinib lead-in period. Results from the safety run-in demonstrated that the combination was well tolerated, and efficacy outcomes were similar to those in the AIM trial, with an ORR of 81%, a CR rate of 62% and a median PFS of 35 months [44]. Another study by Portell et al. investigated whether venetoclax and ibrutinib doses lower than the single-agent dose could achieve similar efficacy with lower toxicities in R/R MCL. Patients initially received venetoclax at 20 mg daily for 1 week prior to initiating ibrutinib, and then continued on a venetoclax dose ramp-up until a maximum daily dose of 200 or 400 mg was reached. Utilizing a continued reassessment method, the findings showed an optimal dose of ibrutinib of 420 mg daily, with venetoclax at 200 mg daily, which is lower than the doses used in the AIM or SYMPATICO trials. Despite the lower doses, the ORR of 83% and a CR rate of 42% were comparable to prior trials [45].

Two monoclonal antibodies that brought about significant improvements in B cell NHL were obinutuzumab and rituximab. Rituximab based induction regimens and maintenance therapy have been shown to improve OS for front-line therapy [4,12]. However, single-agent activity has been limited in R/R MCL and is less impressive than other indolent B cell malignancies, especially after BTKi therapy. Patients with R/R disease, treated with obinutuzumab alone, had a 27% response rate, which was superior to single-agent rituximab [46,47]. In a second trial, patients treated with rituximab and ibrutinib had an ORR of 88%, with a median DOR of 46 months and a 3-year PFS of 54%. High-risk patients (those with high ki67%, the blastoid variant and a high-risk MIPI score) had inferior survival rates, with a median PFS of only 8 months [12,48]. Similarly to venetoclax, there are several studies investigating combinational approaches with these drugs. 

The CDK4/6 inhibitor, palbociclib, has been shown to overcome ibrutinib resistance in MCL cell lines expressing wild-type BTK, by inducing a prolonged early G1 cell-cycle arrest [49]. A phase-I study (PALIBR) evaluated ibrutinib with the addition of palbociclib, with the goal of inducing deeper and longer responses to therapy. This trial demonstrated promising results, with a lower relapse rate than expected with ibrutinib or palbociclib alone. The ORR and CR were 67% and 37%, respectively, with a 2-year PFS of 59.4% and a 2-year DOR of 69.8% [50]. A phase-II trial is underway to validate these findings (NCT02159755).

The phosphoinositide 3-kinase (PI3K) signaling pathway is crucial to many aspects of cell growth, survival and apoptosis, and has been implicated in both the pathogenesis of MCL and a possible pathway of resistance to BTKi. Parsaclisib, a highly selective next-generation PI3K delta (PI3Kδ) inhibitor, showed clinical activity in R/R MCL patients in the phase-II CITADEL-205 trial [51,52]. The study included 108 patients that were BTKi-naïve and 53 patients with prior BTKi exposure. Patients were treated with two different dosing strategies that varied in dosing schedule after week 8 of treatment. The ORR and CR rate were 25% and 2%, respectively, in patients with prior BTKi, while the ORR was 70% in BTKi-naïve patients. The most common adverse events that occurred in >10% of patients included anemia, diarrhea, neutropenia, cough/dyspnea, fatigue, pyrexia and rash [51,52]. Umbralisib, another next-generation PI3Kδ inhibitor, has been investigated in R/R B cell NHL as a monotherapy and in combination with ibrutinib. The combination of umbralisib and ibrutinib was investigated in a phase I/Ib study that included 21 CLL patients and 21 MCL patients with R/R disease [53,54]. Only 2 MCL patients had prior BTKi therapy. In the MCL population, the ORR and CR rate were 67% and 19%, respectively, which is similar to the results reported for ibrutinib monotherapy. The median PFS was 10.5 months, with a 2-year PFS of 49% and an OS of 58%. The most frequent adverse events were diarrhea, infection and transaminitis, with 29% of patients experiencing serious adverse events including atrial fibrillation [53,54]. Longer follow-up studies and additional clinical trials combining these drugs with other agents are ongoing for B cell malignancies, including MCL. 

In a phase I dose-escalation study, the novel antibody drug conjugate, zilovertamab vedotin, was studied in previously treated patients with B cell lymphomas [55,56]. Zilovertamab vedotin comprises zilovertamab, a monoclonal antibody that recognizes extracellular receptor tyrosine kinase-like orphan receptor 1 (ROR1), and the anti-microtubule cytotoxin, monomethyl auristatin E. Of the 51 patients enrolled, 17 had R/R MCL. The ORR was 52.9% for MCL patients: 7 with PR and 2 with CR. The DOR was 7.8 months for all patients. There was a tolerable safety profile, with treatment-related adverse events occurring in 70.6% of patients [55,56]. The ongoing CIRLL study combining zilovertamab (formerly called cirmtuzumab) and ibrutinib included 26 evaluable heavily pretreated MCL patients and showed promising results. The ORR was 81%, with a CR of 35% and a median PFS of 35.9 months. Of the patients included, 45% had intermediate/high MIPI scores and 5 patients had received prior treatment with ibrutinib [57]. These findings support the further development of zilovertamab vedotin as a potential monotherapy or as a combination therapy in patients with R/R MCL.

To date, no single agent has demonstrated superiority after failed BTKi therapy, and not all trials included patients after BTKi failure. The optimal therapy for the management of R/R MCL after BTKi therapy has not been established, and it is likely that combination strategies are needed to provide durable responses in patients, along with a possible bridge to allogeneic HCT or cellular therapies, such as chimeric antigen receptor (CAR) T cells. That being said, although BTKi monotherapy has remained the treatment of choice for relapsed disease, clinical trials may soon support a shift in practice patterns toward the combination of BTKi with other agents in the R/R MCL, which could enhance the durability of response (Table 2). 

## 5. Beyond BTK Inhibitors: Combination Therapies

There have been multiple efforts to combine therapies to induce deeper and more durable responses in R/R MCL, as it is clear that the sequential use of these single targeted agents in the post-BTKi setting has not been as effective (Table 3). In addition, not all trials included patients previously exposed to BTKi; therefore, their value as salvage therapy after BTKi failure remains unclear and additional research in this area is needed. A few notable studies evaluating combination therapies are discussed below. 

A retrospective review of rituximab-bendamustine-cytarabine (R-BAC) after progression on ibrutinib demonstrated an ORR of 83%, with a median PFS of 10.1 months and an OS of 12.5 months. Although these responses were not very durable, 31% of patients were able to successfully bridge to an allogeneic HCT [59]. Therefore, this regimen may be a potential bridge for patients awaiting HCT or CAR T cell therapy. However, challenges exist in treating with this regimen, as dose reductions due to toxicities occurred in 56% of patients, suggesting that the treatment may be too intensive for many patients [59].

The combination of bortezomib and lenalidomide in R/R MCL has been disappointing, with an ORR of 40% [66]. However, the combination of rituximab, bortezomib, lenalidomide, and dexamethasone (DR2IVE) was reported to be generally well-tolerated and was active in a very small population of heavily pretreated MCL patients sequenced after BTKi treatment. A median of 2 cycles was received, with an ORR of 100% in 3 out of 5 patients still alive at the last follow-up, ranging from 3 to 11 months [67]. A trial with a larger sample size is needed to better understand the safety and efficacy of this regimen after BTKi treatment. 

Allogeneic HCT represents a possible therapeutic and even curative option in select patients with R/R MCL; however, its role remains unclear, especially in the era of BTKi and the development of other therapeutic strategies, such as CAR T cell therapy. Currently, in patients where allogeneic HCT is being considered, there is not a defined best strategy for bridging [68]. The MANTLE-FIRST study retrospectively analyzed 55 patients with R/R MCL who underwent allogeneic HCT. The PFS and OS were 53% and 56%, respectively. The incidence of non-relapse mortality was 23% and was higher in patients who were more heavily pretreated (>2 lines therapy) and of older age (>60 years old). The use of BTKi as a bridge did not increase the toxicity rates post-allogeneic HCT [69]. An earlier study that included 22 MCL patients previously treated with ibrutinib, followed by allogeneic HCT, also demonstrated a low non-relapse mortality incidence and a 12-month PFS of over 90% [68]. In selected patients, allogeneic HCT remains an option in R/R MCL, and BTKi such as ibrutinib are a potentially safe and effective option for bridging.

## 6. Beyond BTK Inhibitors: Cellular Therapy—CAR T Cell Therapy

CAR T cell therapy has been a revolutionary cancer breakthrough in otherwise incurable diseases, such as R/R aggressive B cell NHLs and acute lymphoblastic lymphoma, and has been promising after BTKi failure in MCL patients. Brexucabtagene autoleuel (Tecartus) was FDA-approved for the treatment of fit patients with R/R MCL after chemoimmunotherapy and BTKi treatment, based on the results of the ZUMA-2 trial [60]. This study included 74 patients with R/R MCL who were previously treated with up to 5 prior lines of therapy. Prior BTKi treatment included ibrutinib and acalabrutinib. A total of 62% of patients had primary BTKi resistance, while 26% had a relapse after an initial response to BTKi therapy, 7% relapsed after stopping BTKi therapy, and 4% were intolerant of BTKi (mainly ibrutinib) due to its adverse effects. The ORR was 85%, with a CR rate of 59%, and the ORRs remained high among patients despite high-risk disease features including the blastoid variant, TP53 mutation, high MIPI score and a Ki-67 index of 50% or higher. At a median follow-up at 12.3 months, the PFS and OS rates were 61% and 83%, respectively. Common adverse events (grade 3 or higher) included cytopenias (94%), infections (32%), cytokine release syndrome (CRS) (15%) and neurologic events (31%) [60]. In the ongoing phase-I TRANSCEND-NHL-001 trial, lisocabtagene maraleucel (Breyanzi) demonstrated promising clinical activity, with an ORR of 84% and a CR in 19 of 32 R/R MCL patients, with a low incidence of grade 3 or worse cytokine release syndrome and neurological events. There was also a 75% ORR among 12 patients with blastoid morphology, and 58% achieved a CR [70]. CAR T cells have shown impressive activity in R/R MCL, with durable complete responses in a significant percentage of patients [60,70]. Undoubtedly, CAR T cell therapy will play a paramount role in the treatment of R/R MCL after BTKi failure, even in patients with poor prognostic features. However, about 40% of patients will still not respond to CAR T cell therapy [60,70]. A longer follow-up from the ZUMA-2 trial and the results of other clinical trials will help elucidate the exact role that CAR T cell therapy has in the treatment of R/R MCL, and if there is the potential for a possible cure for some patients. 

There is also current interest in combining CART cell therapy with other agents, including BTKi, in an effort to enhance CART efficacy, prolong the duration of remission and maintain negative minimal residual disease. Previous preclinical and clinical trials for B cell neoplasms have demonstrated that CAR T cell therapy, with concurrent ibrutinib, was associated with the improved expansion of CAR T cells and enhancement of the anti-tumor effect, as well as decreased toxicities [71,72,73,74]. The results of the Zuma-2 trial demonstrated similar toxicities to those expected from CAR T cell therapy; however, there was no subgroup analysis to specify whether prior BTKi exposure in patients treated with brexucabtagene autoleucel resulted in similar toxicity rates [60]. In an MCL xenotransplant mouse model, ibrutinib combined with CD19 CAR T cells in vivo led to complete and long-lasting tumor responses [72]. In a study of 7 patients with R/R B cell NHL (3 patients with MCL), with prior poor response to CAR T cell therapy, six patients achieved CR and one achieved a PR, after salvage therapy with repeat CAR T cell therapy, with concurrent ibrutinib. However, patients experienced worse CRS and hematologic toxicities compared to a first treatment with CAR T cell therapy [75]. There is an ongoing phase-II trial assessing the efficacy and safety of tisagenlecleucel (Kymriah) and ibrutinib in R/R MCL (NCT04234061). 

The combination of Bcl-2 inhibitors and CAR T cell therapy has shown promising results in preclinical trials, though not yet in clinical trials for MCL. When tumor cells were pre-sensitized with venetoclax prior to CD19 CAR T cell therapy, there was a significant improvement in the anti-tumor effect of the CAR T cells, as well as enhanced early expansion and long-term persistence of the CAR T cells in vitro [76]. Another study demonstrated that when combining CAR T cells and ABT-737 (navitoclax), another Bcl-2 inhibitor, simultaneously, or as a pre-sensitizer, tumor cell apoptosis was significantly increased [77]. More research is needed to identify the most effective sequence and combination of these agents with CAR T cells in R/R MCL. 

## 7. Beyond BTK Inhibitors: Cellular Therapy—Bispecific Antibodies

Bispecific antibodies (BsAb) comprise two single-chain variable fragments that simultaneously bind to two different antigens: one recognizes an epitope on T cells (typically CD3) and the other recognizes an epitope on the tumor cell of interest. This results in the host’s own immune system attacking the cancer cell through the activation of cytotoxic T cells, leading to the direct T cell-mediated death of the tumor cells [78]. In B cell NHL, ideal antigen targets for malignant cells include CD19, CD20 and CD47. Compared to CAR T cell products, BsAbs have the advantage of being immediately available off the shelf. Below, we discuss the emerging BsAbs that have shown promising anti-lymphoma activity in R/R B cell NHL, including MCL. 

The bispecific t-cell engager (BiTE) with the most evidence to date in B cell malignancies is blinatumomab. Blinatumomab targets both the CD3 subunit on the T cell receptor complex and the CD19 antigen on B cells, which crosslinks T cells and B cells, leading to T cell activation and T cell-mediated lysis of both normal and malignant B cells [61,78]. The phase-I trial MT103-104 investigated blinatumomab in patients with R/R B-NHL with a median of 3 prior treatment lines, including 24 MCL patients. Blinatumomab demonstrated an ORR of 69% and a CR rate of 37% in all patients, with an ORR of 71% in MCL patients [73]. In a follow-up study of 38 patients that included 13 MCL patients, the median OS was 4.6 years, the median PFS was 6.7 months, and the median treatment-free survival (TFS) was 7.6 months [62]. The most frequent AEs reported with blinatumomab were lymphopenia, pyrexia and infections. The most significant toxicity leading to early treatment discontinuation in 13 patients was neurologic. However, the follow-up analysis did not report any long-term neurologic abnormalities secondary to treatment [61,62]. 

Mosunetuzumab is a full-length BsAb that binds CD3 and CD20, to direct T cells to engage and destroy malignant B cells. In contrast to blinatumomab, which is given as a continuous infusion over 4–8 weeks, mosunetuzumab is given in intermittent doses, with initial weekly dosing during the first cycle and then every 21 days for a maximum of 17 cycles [79]. In an ongoing phase-I/Ib dose-escalation trial of R/R NHL patients (270 enrolled patients; 23 MCL patients), mosunetuzumab had an ORR of 37.1%, with a CR rate of 19.4% [63,79]. Interestingly, 30 patients had prior CAR T cell therapy for which the ORR was 38.9%, with a CR rate of 22%. This demonstrates mosunetuzumab’s promising activity in heavily pretreated B cell NHL patients, even with prior CAR T therapy. Mosunetuzumab also had lower rates of CRS (28.9%) and neurotoxicity (43.7%) than was previously observed with blinatumomab [63,79]. Unfortunately, there has not been a subgroup analysis for a histology-specific response, although the results remain seemingly encouraging for R/R MCL.

Glofitamab is a novel Tcell-engaging BsAb with a 2:1 molecular configuration that allows for monovalent binding to CD3 on T cells and bivalent binding to CD20 on B cells. The unique molecular format of glofitamab enables its combination with anti-CD20 antibodies such as obinutuzumab. Studies have demonstrated a promising efficacy, with durable complete responses in heavily pretreated R/R NHL patients, including MCL patients [64,65]. A recent phase-I/II trial included 29 patients with R/R MCL, 69% of whom had prior BTKi therapy, who received 1000 mg or 2000 mg of glofitamab with obinutuzumab pretreatment, prior to glofitamab monotherapy [65]. Patients received either a fixed dosing or step-up dosing schedule of glofitamab. The ORR and complete metabolic response rate (CMR) in patients with prior BTKi therapy were 82.4% and 64.7%, respectively, which was similar in the patients who did not receive prior BTKi therapy (ORR 75% and CMR 75%). CRS (58.6%) and infusion-related reactions (24.1%) were the most common adverse events reported, and all CRS events were grades 1–2, except for one grade-4 CRS event. Neurologic adverse events were grades 1–2 and occurred in 6 patients (20.7%) [65]. Glofitamab as a monotherapy, or used in combination strategies, is a promising new agent for R/R NHL that is relatively well tolerated. 

A recent phase-I trial of REGN1979 or REGENERON, a CD20/CD3 bispecific antibody based on an IgG4 isotype, modified to reduce Fc binding, showed durable responses (a median of 209 days) in R/R B cell NHL, including one patient with MCL [80]. Overall, 96 patients with R/R B cell NHL were included (6 MCL patients), with a median of 3 prior lines of therapy including CAR T therapy. There was no discontinuation due to neurologic complications [80,81]. A phase-II study is currently underway in R/R B cell NHL. Similarly, other BsAbs are being developed and their efficacy is currently being studied in preclinical and phase-I trials, with promising results in the treatment of R/R B cell NHL, including R/R MCL [81]. 

## 8. Conclusions

Over the past decade, there have been exciting developments in MCL that have made substantial improvements in patient outcomes, even those presenting high-risk features. Unfortunately, despite these advancements, many patients still succumb to this aggressive and resistant disease. Once BTKi failure occurs, there remains no standard of care for subsequent treatment. In patients who experience an intolerance of certain BTKi or who develop resistance, switching to a more selective next-generation BTKi, such as LOXO-305, is a reasonable next step. Potential therapies, such as R-BAC or venetoclax, alone or in combination with another agent, have demonstrated favorable short-term responses post-BTKi failure and could be effective as a potential bridge to allogeneic HCT or CAR T cell therapy or in those patients unable to receive these more intensive therapies. Results from the CAR T cell studies have been impressive, and we eagerly await the long-term follow-up of these clinical trials to clarify the durability of remissions. We anticipate the results of more randomized trials, and the development of other targeted therapy will lead to the availability of more effective agents for patients with R/R MCL who have experienced BTKi failure.

## Figures and Tables

**Table 1:** Overview of the data for first- and second-generation BTK inhibitors in R/R MCL.

BTKi	Patients with R/R MCL			ORR	CR	Median PFS
	Number of Patients	Median Lines of Prior Therapy	Patients with High-Risk Features (%) *			
Ibrutinib						
Wang et al., 2015 [14]	111	3	49	67%	23%	13.6 months
Dreyling et al., 2016 [17]	139 **	2	22	72%	19%	14.6 months
Acalabrutinib						
Wang et al., 2019 [20]	124	2	17	81%	43%	20 months
Zanubrutinib						
Song et al., 2020 [24]	68	2	38.4	84%	59%	22.1 months
Tam et al., 2019 [21]	37 ***	1	37.5	84%	22%	18.5 months

* Based on the calculated MIPI score; ** 280 patients were included; 139 were treated with ibrutinib and 141 with temsirolimus; *** 48 MCL patients were included in the study, 37 of whom had R/R MCL.

**Table 2:** Combination strategies to overcome ibrutinib resistance/failure for R/R MCL.

NCT # (Trial Phase)	Drug Combination	N=	ORR	CR	Median PFS	Ongoing Investigation
NCT02471391 (Phase II) [13]	Venetolax + ibrutinib	23	71%	71%	29 months	NCT03112174 (Phase III)
NCT01880567 (Phase II) [12]	Rituximab + ibrutinib	50	88%	40%	3 year−54%	--
NCT02159755 (Phase I) [46]	Obinutuzumab + venetoclax + ibrutinib	12	66.6%	25%	Not reached	--
NCT02460276 (Phase II) [58]	Lenalidomide + Rituximab + ibrutinib	50	76%	56%	Not reached	--
NCT02159755 (Phase I) [50]	Palbociclib + ibrutinib	27	67%	37%	2 year−59.4%	NCT02159755 (Phase II)
NCT02268851 (Phase I/Ib) [53]	Umbralisib + ibrutinib	21	67%	19%	10.5 months	NCT02268851 (Phase II)

**Table 3:** Available treatment for R/R MCL and after BTK inhibitor failure.

	Data in all R/R MCL Patients				Data in Patients with Prior BTKis			
	N=	ORR	CR	Median PFS	N=	ORR	CR	Median PFS
LOXO-305 (pirtobrutinib) [29,31]	56	52%	23%	NR *	52	52%	NR *	NR *
Bortezomib [35]	155	33%	8%	9.2 months	--	--	--	--
Temsirolimus [37]	170	22%	2%	4.8 months	--	--	--	--
Lenalidomide Monotherapy [38,40]	54	78%	19%	8.7 months	13	15%	0%	NR *
Venetoclax [41,42,43]	28	75%	21%	11.3 months	20	53%	18%	3.2 months
R-BAC [6,59]	20	80%	75%	NR *	36	83%	N/A	10.1 months
Parsaclisib [51,52]	108	70%	15.2%	11.1 months	53	25%	2%	3.7 months
Brexucabtagene autoleuel [60]	--	--	--	--	74	85%	59%	12.3 months −61%
Blinatumomab [61,62]	24	71.1%	37% **	6.7 months **	--	--	--	--
Mosunetuzumab [63]	23	37.1% **	19.4% **	NR *	--	--	--	--
Glofitamab [64,65]	4	75%	75%	NR	17	82.4%	64.7%	NR

* Data not reported; ** denotes data for total NHL population instead of only MCL patients.
